# Mulches Used in Highbush Blueberry and Entomopathogenic Nematodes Affect Mortality Rates of Third-Instar *Popillia japonica*

**DOI:** 10.3390/insects12100907

**Published:** 2021-10-05

**Authors:** Justin M. Renkema, Jean-Philippe Parent

**Affiliations:** 1 London Research and Development Centre—Vineland Campus, Agriculture and Agri-Food Canada, 4902 Victoria Ave N, Vineland Station, ON L0R 2E0, Canada; 2 Centre de Recherche et Développement de Saint-Jean-sur-Richelieu, Agriculture and Agri-Food Canada, 430 Boul Gouin, Saint-Jean-sur-Richelieu, QC J3B 3E6, Canada; jean-philippe.parent2@agr.gc.ca

**Keywords:** Japanese beetle, *Heterorhabditis bacteriophora*, *Steinernema scarabaei*, biological control, compost, woodchips, sawdust

## Abstract

**Simple Summary:**

Japanese beetle is a serious pest of many crops, including blueberry. While the adult beetles feed on leaves, the grubs (immatures) feed on plant roots and can cause wilting and reduced plant growth. Farmers rely on insecticides to control Japanese beetle, but nematodes that attack and kill grubs in the soil may be as effective as insecticides in some cases. Mulches of compost, woodchips, sawdust, and tree bark are commonly used in blueberry production, but it is not known how mulches affect Japanese beetle grubs or the effectiveness of beneficial nematodes to control grubs. We placed grubs in soil beneath different mulches in the laboratory and tested two nematodes. The species *H. bacteriophora* killed almost all grubs in all mulches, but *S. scarabaei* was not as effective and was more affected by mulch type. A mulch of compost + woodchips and sawdust caused 60% grub mortality without adding nematodes. In a field experiment during October, the nematodes caused 50% grub mortality, which was lower than expected and likely due to cool soil temperatures. We recommend using *H. bacteriophora* for Japanese beetle grub management in blueberry, regardless of the mulch type being used.

**Abstract:**

*Popillia japonica* Newman (Japanese beetle) is an invasive, polyphagous pest in North America, as adults feed on plant foliage and larvae on roots. Management in crops relies on foliar and soil applications of insecticides, but entomopathogenic nematodes (EPN) are effective biocontrol agents. In highbush blueberry, mulches (composts, woodshavings, sawdust, bark) are used for weed control and fertility. Therefore, our objective was to determine the effects of *Heterorhabditis bacteriophora* and *Steinernema scarabaei* on third-instar *P. japonica* in substrates commonly used as mulches in blueberry. In containers in the laboratory, larval mortality was 90–100% with *H. bacteriophora* for all substrates, but rates with *S. scarabaei* were lower and variable among substrates. A mixture of municipal compost + woodchips/sawdust resulted in 60% larval mortality without adding EPN, but few nematodes were recovered, indicating other causes of death. In a field microplot experiment in October, larval mortality rates were 50% at most for all EPN and substrate type combinations, likely due to lower than optimal soil and substrate temperatures for EPN survival and infectivity. Overall, a compost and woodchip/sawdust mulch should help suppress *P. japonica* populations in blueberry, and applying *H. bacteriophora* when temperatures are optimal to mulches can provide excellent larval control.

## 1. Introduction

*Popillia japonica* Newman (Coleoptera: Scarabaeidae) (Japanese beetle) is a pest of numerous agricultural and horticultural crops in Canada, USA, Portugal, Italy, Switzerland, Russia and Japan, including blueberry (*Vaccinium* spp.) [1,2]. Adult beetles congregate on host plants and feed on foliage for about a month, and, after eggs laid in the soil hatch, larvae feed and develop on roots [3]. While larvae are typically turfgrass or perennial grass pests, tree nurseries also suffer from *P. japonica* [3,4]. In highbush blueberry (*Vaccinium corymbosum*), particularly in young plantings in light textured soil, stunted growth and reduced plant vigor are symptoms of larval root feeding, and extensive feeding causes poorly-rooted bushes that can be easily pulled from the soil [5]. Soil applied insecticides, such as halofenozide, imidacloprid, and other neonicotinoids, were effective against *P. japonica* larvae in grass and field tree nurseries [6,7]. In blueberry in Canada, only imidacloprid is registered for soil application against *P. japonica* and other scarab beetle larvae.

Entomopathogenic nematodes (Rhabditida: Steinernematidae and Heterorhabditidae) (EPN) have been assessed against soil-dwelling life stages of agricultural pests, and several species are effective against *P. japonica* [8,9]. *Heterorhabditis bacteriophora*, *Heterorhabditis zealandica*, *Steinernema glaseri* and *Steinernema scarabaei* all caused greater than 80% mortality of third-instar *P. japonica* in soil in controlled environments after 14 days [10,11]. However, in turfgrass, *H. bacteriophora* was effective (>80% mortality) against first- and second-instars but not against third-instars [12], and morality was variable (34–97%) in another series of outdoor experiments [13]. In highbush blueberry, *S. scarabaei* reduced *Anomala orientalis* (Waterhouse) (Coleoptera: Scarabaeidae) by over 80%, with applications made to bare soil around bushes [14]. Therefore, there is good potential for EPN control of *P. japonica* in blueberry, but efficacy may depend on weed management, fertility sources, and other horticultural practices affecting the soil.

Mulching with pine bark, pine needles, hardwood chips, leaves, or sawdust is a common practice for weed management in highbush blueberry, particularly in organic production [15,16,17]. From a recent survey, 7 of 8 highbush blueberry growers in Ontario, Canada, applied a mulch every 2–3 years of chipped wood pallets, sawdust with manure from horse barns, pine bark, sawdust, wood shavings or discarded peat moss [18]. Using compost or a compost and mulch mix also improved plant growth and yield [17], provided pest control, and benefitted natural enemies [19,20]. Composts may also improve the virulence of and pest mortality rates caused by EPNs compared to certain soil types [21], although immaturely-cured compost may negatively affect EPN virulence [22]. In general, application of EPNs into composts, mulches, or other soil amendments rather than soil may protect EPNs from UV damage and buffer them from temperature and moisture extremes while promoting EPN movement and contact with pests [8].

As highbush blueberry plants can be negatively affected by *P. japonica* root feeding and imidacloprid is the only registered insecticide for soil application against *P. japonica* in blueberry in Canada, additional tools for larval control are needed to develop an integrated management strategy for this pest. The goal of the research was to provide knowledge on whether substrates commonly used as mulches in highbush blueberry production impacted mortality rates of *P. japonica* and whether entomopathogenic nematode efficacy was affected by substrate type. Our specific objective was to determine the effects of aqueous preparations of commercial *H. bacteriophora* and *S. scarabaei* strains applied to organic substrates (woodchips and composts) on the mortality rates of third-instar *P. japonica*. Our results will be useful in advancing a management plan for *P. japonica* in blueberry, with control tactics that target not only adults but also larvae.

## 2. Materials and Methods

### 2.1. Laboratory Experiment

Third-instar *P. japonica* were removed by hand from overturned sod at the Agriculture and Agri-Food Canada (AAFC) research farm near Jordan Station, ON, Canada (43.175680, −79.360977) on 19 and 22 October 2018. Larvae were stored individually in plastic containers (60 mL) with moist sand at 4 °C.

Clear, plastic containers (1 L; 11 cm top diameter; 15 cm height) with three small holes in the bottom were partially filled with sieved, double-autoclaved soil (250 mL) from the same area at the AAFC farm where the larvae were collected. A mix of creeping red fescue, perennial rye and Kentucky bluegrass (Scotts^®^ Turf Builder^®^ Grass Seed Shady Areas Mix) was grown from seed (1 g per container) in the soil in the containers for 4 weeks in a greenhouse and watered as needed. A day before the start of the experiment, the grass was cut at the soil level and clippings removed from the container. Grass roots were a food source for the larvae.

Larvae were removed from cold storage and held at room temperature for 24 h before placing a healthy larva in a container in a small depression made in the soil. Larvae that did not burrow into the soil within 1 h were replaced with another larva. On the same day, one of the following substrates (500 mL) was added to each container: woodchip and sawdust mixture (1:1 by volume), woodchip and sawdust mixture + horse manure compost (1:1 by volume), woodchip and sawdust mixture + municipal compost (1:1 by volume), or soil (150 mL). Woodchips and sawdust were from food-grade softwood pallets and obtained from outdoor piles at a blueberry farm near Lowbanks, Ontario. Horse manure compost was from outdoor piles at a blueberry farm near Dundas, Ontario. Municipal compost was organics from the Green Bin program (Lifecycle™, AIM Environmental Group, Stoney Creek, ON, Canada).

On the day after placing mulches and larvae in containers, EPN treatments of *S. scarabaei* (Evergreen Bio Innovations Ltd., Ajax, ON, Canada) and *H. bacteriophora* (Natural Insect Control, Stevensville, ON, Canada) were prepared in cool, distilled water according to the manufacturer’s instructions. Stock solutions were diluted to a rate of 50 infective juveniles (IJs)/cm^2^, and aliquots of 40 mL were poured from small plastic containers (120 mL) over the surface of the mulch or soil in each container. Containers were arranged in a completely randomized design and incubated in a growth chamber at 20 °C. There were nine replicates of each substrate type by EPN combination by incubation time period combination. 

After 10 or 20 days in the growth chamber, the containers were removed and overturned on a plastic tray. The contents were separated into the mulch and soil components and searched to recover larvae. Larvae were recorded as dead or alive; moribund larvae and larvae that were not found were considered dead. For the 10-day containers, dead and moribund larvae were placed individually in white traps. Because most (>95%) of the *P. japonica* larvae were found in the soil beneath the substrates, samples of the soil (25 g) and not the substrates were collected and placed in Baermann trays. White traps and Baermann trays were held at room temperature (20–23 °C) and checked daily to rewet the filter paper if necessary. After one week, the water in the white traps or Baermann trays was collected into test tubes, and numbers of nematodes from cadavers were counted in gridded dishes at 30–40× magnification under a stereomicroscope.

### 2.2. Microplot Field Experiment

In early October 2019, microplots, galvanized steel cubes (30 × 30 × 30 cm) open on opposite ends, were placed into holes dug into a tilled area at the AAFC research farm. Microplots were spaced one meter apart, in a 10 × 10 grid with a total area of about 100 m^2^. Black landscape fabric was taped over the bottom, open ends of the microplots before they were placed in the holes, and the top of the microplots was about 5 cm higher than the surrounding soil surface. Microplots were backfilled with soil. Tested substrates were: (1) a mixture (1:1) of moistened sand and peat, (2) field soil from a depth of 5–20 cm at the AAFC research farm and sieved through a 2.5 × 2.5 cm screen, (3) woodchips 1 (same source as for the laboratory experiments) dug from piles on 24 September 2019, (4) woodchips 2 from eastern white pine (*Pinus strobus* L.) and dug from piles at a blueberry farm near Frogmore, Ontario, on 9 October 2019, (5) a mixture (1:1) of municipal compost (same source as for laboratory experiments) and woodchips. Each substrate was placed in a pile on and covered with tarps next to the microplot area. Water was added to the piles and mixed into the substrates until they were wet but not dripping. On 10 October 2019, each microplot was partially filled with 8 L of a substrate. The substrate was tamped down by hand so that the surface was about 5 cm below the top of the microplot.

On 10 and 11 October 2019, third-instar *P. japonica* were removed from overturned sod at the AAFC research farm and placed (three each day) on the surface of the substrate in each microplot. Larvae that did not burrow within 1 h into the substrate were replaced with healthy larvae. In the evening of 11 October 2019, aqueous preparations (200 mL per microplot) of *S. scarabaei* and *H. bacteriophora* (same sources, preparation methods and rates as in the laboratory experiment) were poured from plastic beakers evenly over the surface of the substrate in each microplot, with water as a control. Substrate type and EPN species combinations were arranged in a completely randomized block design with seven replicates for the mulch and EPN combinations and six replicates for the substrate and control combinations.

On 30 October 2019, the soil around each microplot was dug out and the microplot was lifted out of the hole on a spade. Substrates were hand-sorted on tables in the field, and larvae were recorded as dead or alive; moribund larvae and larvae that were not found were considered dead. Nematodes were extracted and counted from dead and moribund larvae as in the laboratory experiment. Substrate samples were collected in the field into plastic bags from four of the replicates. Subsamples (50 g), stored at 4 °C for one week, were placed in a Baermann trays and nematodes counted as in the laboratory experiment.

### 2.3. Statistical Analysis

For the laboratory experiment, a generalized linear model with a binomial distribution (dead or alive) and a logit function was used to determine whether there were significant effects of substrate type, EPN species, and incubation time on larval mortality. An initial model including the three factors and all their interactions resulted in non-significance of all interactions that included incubation time (*p* > 0.05). Therefore, the data was subsequently analyzed separately by incubation time, with substrate type, EPN species and their interaction as factors. For the counts of EPNs from cadavers and substrate samples, an ANOVA was used to determine effects of EPN species and substrate types. For the *P. japonica* experiment, counts were log_10_ + 1 transformed to normalize the distributions of the data and residuals. Back-transformed means and 95% confidence intervals (CI) are reported.

For the outdoor microplot experiment, a mixed-model ANOVA was used to determine the effects of substrate type and EPN species and their interaction on total larval mortality and nematode recovery from mulch samples, with block included as a random effect. Residuals were normally distributed and no transformation was necessary for mortality data. Numbers of nematodes recovered from mulch were log_10_ transformed to normalize error variance. For nematode recovery from cadavers, each cadaver was considered an experimental unit, and the effects of mulch type and EPN species were tested with separate ANOVAs on numbers of nematodes recovered; no data transformation was necessary. All statistical tests were done using JMP^®^ 15.0.0 software [23] at α = 0.05.

## 3. Results

### 3.1. Laboratory Experiment

**Figure 1:**
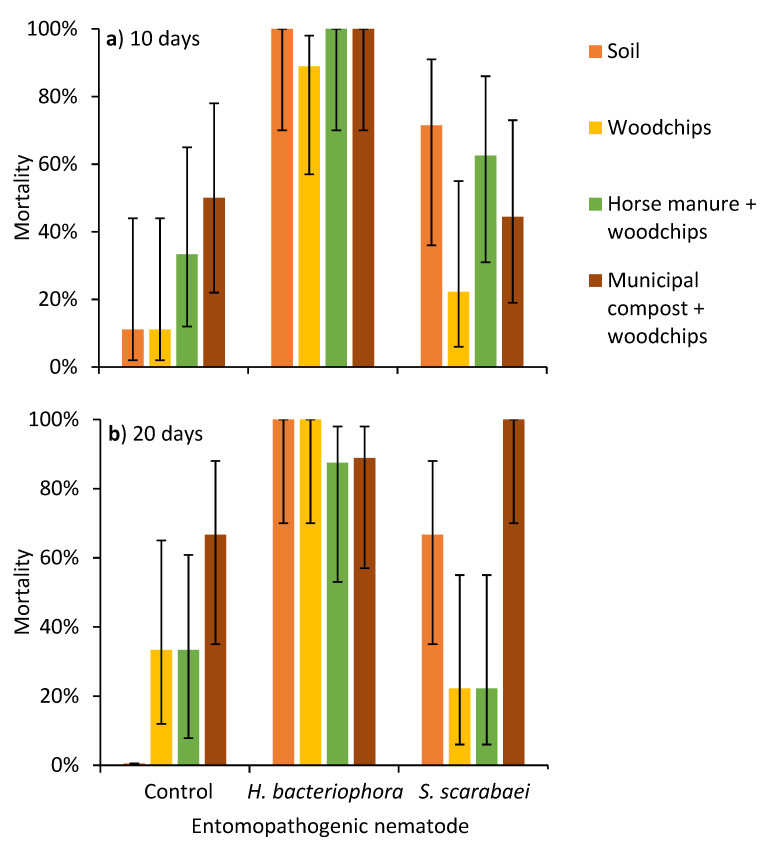
Mean (± 95% CI) mortality of third-instar *Popillia japonica* at (**a**) 10 and (**b**) 20 days at 20 °C after aqueous preparations (40 mL) of the entomopathogenic nematodes *Heterorhabditis bacteriophora* or *Steinernema scarabaei* (50 infective juveniles/cm^2^) were added to soil, woodchips, woodchips, and horse manure or wood chips and municipal compost in plastic containers (1 L).

After incubation at 20 °C for 10 days, there was an effect of EPN species (χ^2^ = 49.1; df = 2; *p* < 0.0001), but not substrate type (χ^2^ = 6.4; df = 3; *p* = 0.094) or their interaction (χ^2^ = 5.3; df = 6; *p* = 0.502) on *P. japonica* mortality (Figure 1a). After 20 days, there was an effect of EPN species (χ^2^ = 35.1; df = 2; *p* < 0.0001) and the EPN species by substrate type interaction (χ^2^ = 20.0; df = 6; *p* = 0.003) on *P. japonica* mortality, but there was not an effect of substrate type (χ^2^ = 7.1; df = 3; *p* = 0.069) (Figure 1b).

**Figure 2:**
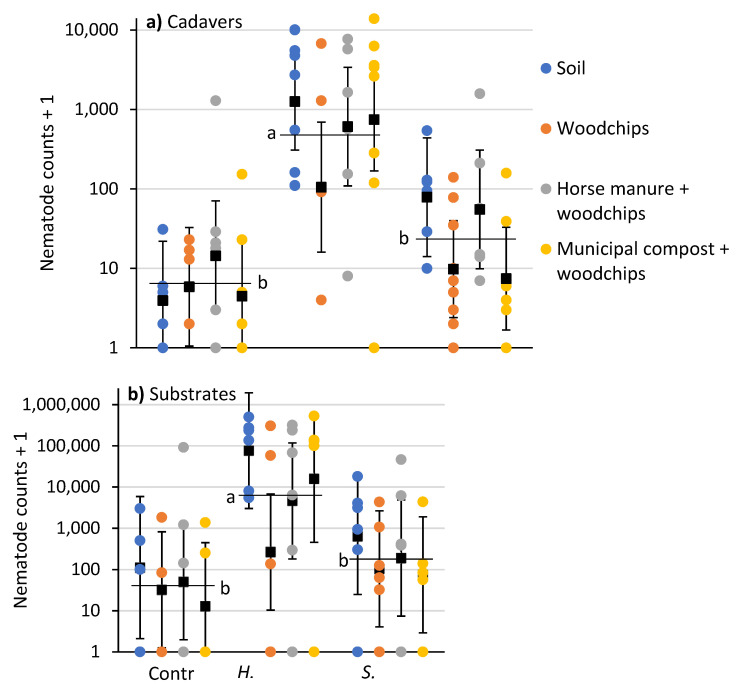
Counts (colored circles) and means (±95% CI) (black squares) of nematodes recovered in white traps from (**a**) *P. japonica* cadavers and (**b**) soil samples beneath substrates 10 days after aqueous preparations (40 mL) of *Heterorhabditis bacteriophora* or *Steinernema scarabaei* (50 infective juveniles/cm^2^) were added to soil, woodchips, horse manure and woodchips or municipal compost and woodchips in plastic containers (1 L). Entomopathogenic nematode species means (horizontal black lines) with the same letter are not significantly different in each panel (Tukey’s HSD test, α = 0.05).

After 10 days, the numbers of nematodes recovered from *P. japonica* cadavers was affected by EPN species (*F* = 29.8; df = 2, 71; *p* < 0.0001), but not substrate type (*F* = 2.2; df = 3, 71; *p* = 0.092) or their interaction (*F* = 1.0; df = 6, 71; *p* = 0.426). Numbers of nematodes recovered from soil samples was affected by EPN species (*F* = 9.5; df = 2, 56; *p* = 0.0003), but not substrate type (*F* = 1.5; df = 3, 56; *p =* 0.217) or their interaction (*F* = 0.5; df = 6, 56; *p =* 0.769). There were more nematodes recovered from cadavers and substrate samples from containers with *H. bacteriophora* than with *S. scarabaei* or controls (Figure 2).

### 3.2. Microplot Field Experiment

**Figure 3:**
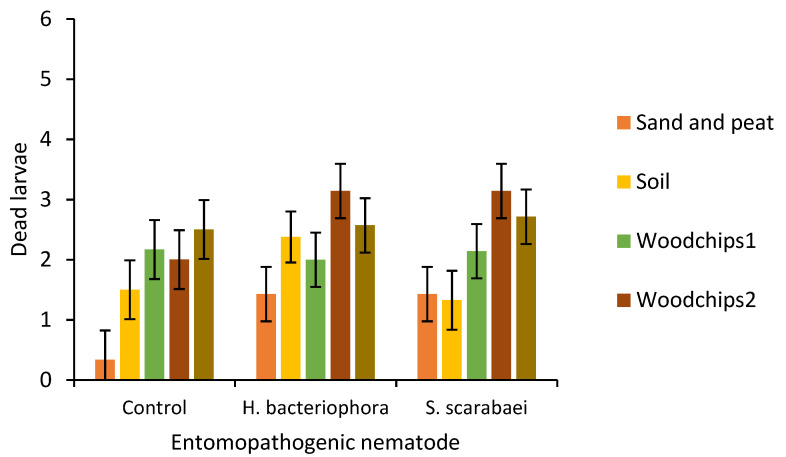
Number of dead *Popillia japonica* larvae (mean ± SEM) 20 days after placing six third-instars per outdoor microplot (30 × 30 × 30 cm) that was partially filled with either sand and peat, field soil, woodchip types 1 or 2, or municipal compost and woodchips type 1 and inoculated with the entomopathogenic nematodes *Heterorhabditis bacteriophora* or *Steinernema scarabaei* (50 infective juveniles/cm^2^). Galvanized steel microplots were placed in the soil in Agriculture and Agri-Food Canada research plots near Jordan Station, ON, Canada.

After 20 days, there was a significant effect of substrate type (*F* = 6.6; df = 4, 79.43; *p* < 0.0001) but not EPN species (*F* = 2.2; df = 2, 81.73; *p =* 0.121) or their interaction (*F* = 0.8; df = 8, 79.65; *p =* 0.628) on larval mortality (Figure 3). Larval mortality was significantly higher in plots with woodchips 2 (2.8 ± 0.27) and woodchips 1 + municipal compost (2.6 ± 0.27) than in plots with sand and peat (1.1 ± 0.27).

**Figure 4:**
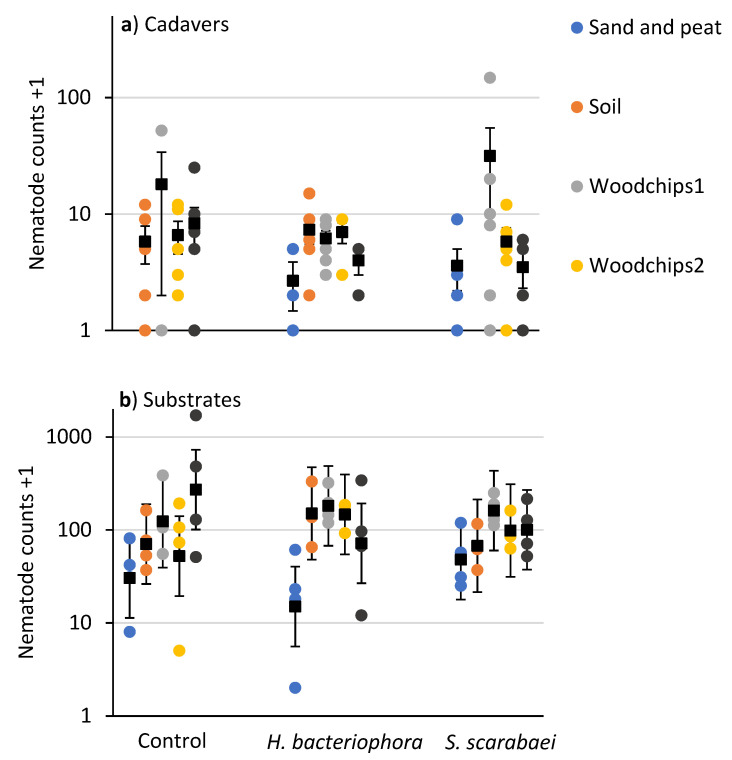
Counts (colored circles) and means (±SEM in (**a**) and ± 95% CI in (**b**)) (black squares) of nematodes recovered in white traps from (**a**) *Popillia japonica* cadavers and (**b**) mulch samples taken 20 days after inoculation of outdoor microplots (30 × 30 × 30 cm) partially filled with sand and peat, field soil, woodchip type 1 or 2, or municipal compost and woodchips type1 and inoculated with *Heterorhabditis bacteriophora* or *Steinernema scarabaei*. Galvanized steel microplots were placed in the soil in Agriculture and Agri-Food Canada research plots near Jordan Station, ON, Canada. No cadavers were recovered from the sand and peat control and the soil plus *S. scarabaei* microplots.

Numbers of EPNs extracted from *P. japonica* cadavers were relatively low (compared to the laboratory experiment) and not affected by substrate type (*F* = 1.3; df = 4, 55; *p* = 0.265) or EPN species (*F* = 0.7; df = 2, 55; *p* = 0.497) (Figure 4a). The number of EPNs extracted from substrates differed due to substrate type (*F* = 5.6; df = 4, 38.3; *p =* 0.001) but not due to EPN species (*F* = 0.02; df = 2, 38.2; *p* = 0.98) or their interaction (*F* = 1.4; df = 8, 38.1; *p* = 0.227) (Figure 4b). Significantly fewer EPNs were recovered from the sand and peat mixture (27.9 ± 0.30) than the woodchips 1 (153.4 ± 0.31), woodchips 2 (91.0 ± 0.31), and municipal compost + woodchips 1 (124.8 ± 0.30) substrates.

The mean daily morning temperature differed due to substrate type (*F* = 5.22; df = 4, 144; *p* = 0.0006), and was significantly greater, by about 2 °C, in municipal compost + woodchips 1 than other substrate types (Table 1). The mean daily afternoon temperature also differed due to substrate type (*F* = 5.69; df = 4, 145; *p* = 0.003), and was significantly greater, by about 2 °C, in municipal compost + woodchips 1 than both types of woodchips (Table 1). The maximum and minimum temperatures recorded ranged from about 15–18 °C and 6–9 °C, respectively, among substrate types (Table 1). 

## 4. Discussion

Our laboratory results showed that *H. bacteriophora* caused consistently high mortality of third-instar *P. japonica* regardless of substrate type, but mortality rates with *S. scarabaei* tended to be lower and more variable among substrate types. Moderate levels of third-instar *P. japonica* mortality occurred in substrates without EPN (60% after 20 days with municipal compost and woodchips), but few nematodes were extracted from the cadavers or the soil beneath the substrates, indicating primarily other causes of larval death. In the field microplots, *P. japonica* larval mortality was 50% at best, and few nematodes were found in cadavers or substrates. Where root-feeding by *P. japonica* larvae occurs in highbush blueberry, applications of *H. bacteriophora* should provide nearly complete control regardless of substrate or mulch type. In addition, mulches of compost mixed with woodchips should also provide some suppression of *P. japonica* populations.

We expected high levels of third-instar *P. japonica* mortality with *S. scarabaei*, as larval mortality in a previous study was nearly 100% at application rates that were half of what we used [11]. In our experiment, mortality due to *S. scarabaei* was 70% in soil and lower in substrates (except in municipal compost + woodchips after 20 days). Soil moisture affected *S. scarabaei* virulence, resulting in 40% and 20% third-instar *P. japonica* mortality in the wettest and driest soils tested, respectively [24], and the compost and woodchip containing substrates had 2–3× more moisture by weight than soil in this experiment (data not shown). However, the substrates were less dense than soil, meaning that the EPN may have experienced a drier environment in the substrates than in soil, particularly in woodchips with large pores that presumably resulted in more rapid drying than other substrates. In a previous study, potting mix reduced mortality rates of *A. orientalis* to 30% compared to 80% in all tested soil types [25]. Exactly how low density substrates with high organic matter affect EPN species is not known, although highly basic or acidic solutions caused low survival of EPN infective juveniles, particularly some strains of *H. bacteriophora* [26]. However, in the laboratory experiment, we found 10X fewer nematodes on average in the soil beneath substrates than in soil only, indicating *S. scarabaei* may not have been able to efficiently move through the substrates. Future research on *S. scarabaei* and physical properties of organic substrates is needed so that predictions about its efficacy in non-soil environments can inform its usefulness as a biological control agent.

In both experiments, *P. japonica* larval mortality in controls (without added EPN) tended to be lower in soil or sand and peat than in substrates, and reached 60% in the laboratory in municipal compost + woodchips. However, it was not clear that the cause of death was from infection by EPN occurring naturally in the substrates, as low numbers of nematodes were recovered from cadavers and the substrates. Cadavers from *H. bacteriophora* treatments had 10–100× more nematodes than cadavers in controls. Larval *P. japonica* may be infected by several fungal and bacterial pathogens, including *Paenibacillus popilliae*, *P. lentimorbus*, an isolate of *Bacillus thuringiensis*, *Metarhizium anisopliae*, and *Beavuaria bassiana* [3]. Suppressive effects of beneficial microbiota (e.g., *Bacillus* spp.) in composts on plant pathogens and disease have been studied, but less is known about the effects of organic soil amendments on crop pests [27]. Further research on insect pathogens in general and disease-causing agents of *P. japonica* and white grubs specifically in composts and other materials added to crops is needed. Mulches may also directly affect *P. japonica*, as a high density of coconut fiber was an effective physical oviposition barrier in containers with grape plants [28].

In the field microplot experiment, lower than optimal temperatures for *H. bacteriophora* and *S. scarabaei* infectivity were the likely cause of minimal effects on *P. japonica* larvae. *Heterorhabditis bacteriophora* was moderately to not-at-all effective against third-instar *P. japonica* when used in autumn at mean soil temperatures less than 15 °C [29,30], and *S. scarabaei* was ineffective against *E. orientalis* at laboratory temperatures less than 15 °C [31]. The mean afternoon temperature was nearly 15 °C in the municipal compost + woodchips plots, higher than in other substrates, and *P. japonica* larval mortality rates tended to be higher in municipal compost + woodchips than other substrates, except for woodchips 2. Using EPN against *P. japonica* in autumn rather than spring may be advantageous because pre-overwintering third-instars are more susceptible to EPN than post-overwintering third-instars [32], and a portion of the population may be second-instars that are more susceptible to EPN than third-instars [12]. However, even though some substrates can increase temperature compared to soil, EPN applications should be made at least a few weeks earlier than in this experiment (10–30 October) when soil temperatures should be higher and not limit effectiveness against *P. japonica* larvae.

Shifting management of *P. japonica* in blueberry from reliance solely on insecticide applications when adults cause defoliation to sustainable approaches that also target larvae will require continued research. In young highbush blueberry plantings where larval root-feeding is a concern, an application of a compost and woodchip/sawdust mix in late summer when *P. japonica* larvae are already in the soil followed by an application on *H. bacteriophora* should provide excellent control. In the following seasons, since mulch is not typically applied every year, *P. japonica* oviposition in mulches around blueberry bushes and vertical movement of larvae to roots [33] will need to be assessed to determine the feasibility and efficacy of an EPN application. Once blueberry plantings are established, row middles planted with grasses are likely to harbor most of the larval *P. japonica* population. Middles can be tilled to kill larvae and effectively reduce *P. japonica* populations [34], but in cases where tillage is not appropriate, a strategy for and the economics of applying EPN should be investigated. In conclusion, mulching in highbush blueberry for weed control and greater plant productivity should also serve to suppress *P. japonica* populations, benefit EPN infectivity rates, and thereby reduce foliar insecticide use against adults.

## Figures and Tables

**Table 1:** Mean and daily maximum and minimum (±SEM) temperatures recorded 5 cm below the surface of mulches at 0900 h (morning) and 1500 h (afternoon) on 10 of 14 days (actual dates) of an outdoor microplot experiment at Agriculture and Agri-Food Canada research plots near Jordan Station, Ontario. Means within columns with the same letter are not significantly different (Tukey’s HSD test, α = 0.05).

Mulch Type	Morning (°C)	Afternoon (°C)	Maximum (°C)	Minimum (°C)
Sand and peat	08.7 ± 0.41 b	13.7 ± 0.43 ab	18.1 ± 0.62 (21 October)	6.0 ± 0.10 (29 October)
Soil	08.8 ± 0.39 b	13.3 ± 0.35 ab	15.9 ± 0.27 (21 October)	6.7 ± 0.18 (29 October)
Woodchips 1	09.2 ± 0.38 b	12.7 ± 0.33 b	15.9 ± 0.52 (21 October)	6.6 ± 0.19 (29 October)
Woodchips 2	08.9 ± 0.39 b	12.4 ± 0.36 b	15.3 ± 0.43 (21 October)	7.1 ± 0.09 (18 October)
Municipal compost + woodchips 1	10.9 ± 0.41 a	14.6 ± 0.32 a	17.4 ± 0.10 (22 October)	8.8 ± 0.09 (18 October)

## Data Availability

The data presented in this study are available on request from the corresponding author.
